# Bilateral primary vitreoretinal lymphoma masquerading as fungal endophthalmitis- a case report

**DOI:** 10.1186/s12348-024-00426-w

**Published:** 2024-12-30

**Authors:** Manisha Agarwal, Alankrita Muralidhar, Tanya Jain, Prashant Katre, Arpan Gandhi, Priyanka Gupta, Charu Gupta, Shishir Narain

**Affiliations:** 1 Dr Shroff’s Charity Eye Hospital, 5072, Kedarnath Road, Daryaganj, New Delhi 110002 India; 2 Shroff Eye Center, Kailash Colony, New Delhi, India

**Keywords:** Primary Vitreoretinal Lymphoma (PVRL), Spectral Domain Optical Coherence Tomography (SD-OCT), Pars Plana Vitrectomy (PPV), Retinal Pigment Epithelium (RPE), Interleukin (IL)

## Abstract

**Purpose:**

To report a case of bilateral primary vitreoretinal lymphoma (PVRL) masquerading as endophthalmitis in a patient with a history of bilateral cataract surgery and COVID-19.

**Observation:**

A 60-year-old male patient presented with diminution of vision in both the eyes. There was a history of bilateral cataract surgery done 2 months back at a gap of one week and COVID-19 infection treated with high dose systemic corticosteroids. Patient presented with dense vitritis with yellowish sub-retinal pigment epithelium (RPE) deposits in both the eyes six weeks after cataract surgery. It was clinically suspected to be endophthalmitis. Diagnostic vitrectomy was done in both the eyes and vitreous sample was negative on microbiological and cytological examination. Multimodal imaging along with sub retinal biopsy helped in confirming the diagnosis. Management was done using multiple intravitreal methotrexate injections and remission was achieved.

**Conclusion:**

Vitritis with sub-RPE yellowish deposits may be mistaken for infectious endophthalmitis specially in a patient with a history of intraocular surgery or immunosuppression. PVRL is a great masquerader and is to be kept in mind while diagnosing a middle-aged patient with infectious or non-infectious uveitis.

## Introduction

Primary vitreoretinal lymphoma (PVRL) is a life-threatening lymphocytic neoplasm. It is a great mimicker of non-infectious uveitis, as it often presents with vitritis which shows a good response to corticosteroids but has recurrence on stopping them [1, 2].

We report a case of PVRL mimicking infectious uveitis in a patient with a history of an intraocular surgery in the recent past and a history of immunosuppresion secondary to COVID-19 infection treated with high dose oral corticosteroids. This case highlights that rarely PVRL may also mimic endophthalmitis and therefore we need to keep it in mind specially in middle aged patients.

## Case report

A 60-year-old male patient presented with blurring of vision in both the eyes for last 15 days He had been diagnosed as having intermediate uveitis elsewhere with multiple recurrences and treated with oral corticosteroids. He had developed secondary glaucoma and complicated cataract secondary to corticosteroids. He had undergone cataract surgery with intraocular lens implantation in both the eyes 2 months back, one week apart. He was on topical steroids and anti-glaucoma medication. He was a known case of diabetes, hypertension, and coronary artery disease for last 20 years. There was a past history of receiving high dose systemic steroids and admission in intensive care unit for COVID-19 infection.

Post cataract surgery in both the eyes he was doing well however 6 weeks later he developed blurring of vision with bilateral dense vitritis and hazy view of yellowish sub-retinal deposits resembling exudates. He was suspected to have endophthalmitis which could be exogenous or endogenous. Urine and blood cultures were negative. He underwent a bilateral pars plana vitrectomy (PPV) along with intravitreal vancomycin (1 mg/0.1 ml), voriconazole (50цgm/0.1 ml) and moxifloxacin (500цgm/0.1 ml). Post operatively he was treated with topical and oral voriconazole, oral cefuroxime, topical anti-glaucoma medications -0.2% brimonidine tartrate and 0.5% timolol maleate twice a day.

**Fig. 1:**
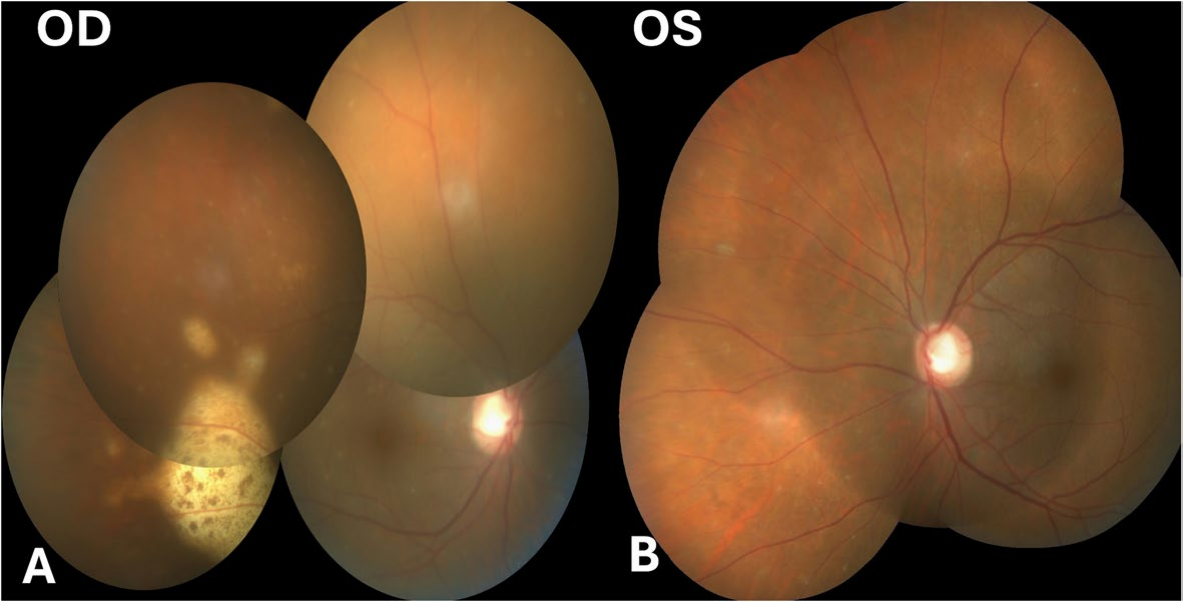
Fundus photograph of the right eye showing a sub retinal yellowish lesion and a very small yellowish leison at the macula of the left eye along with few hypopigmented spots

**Fig. 2:**
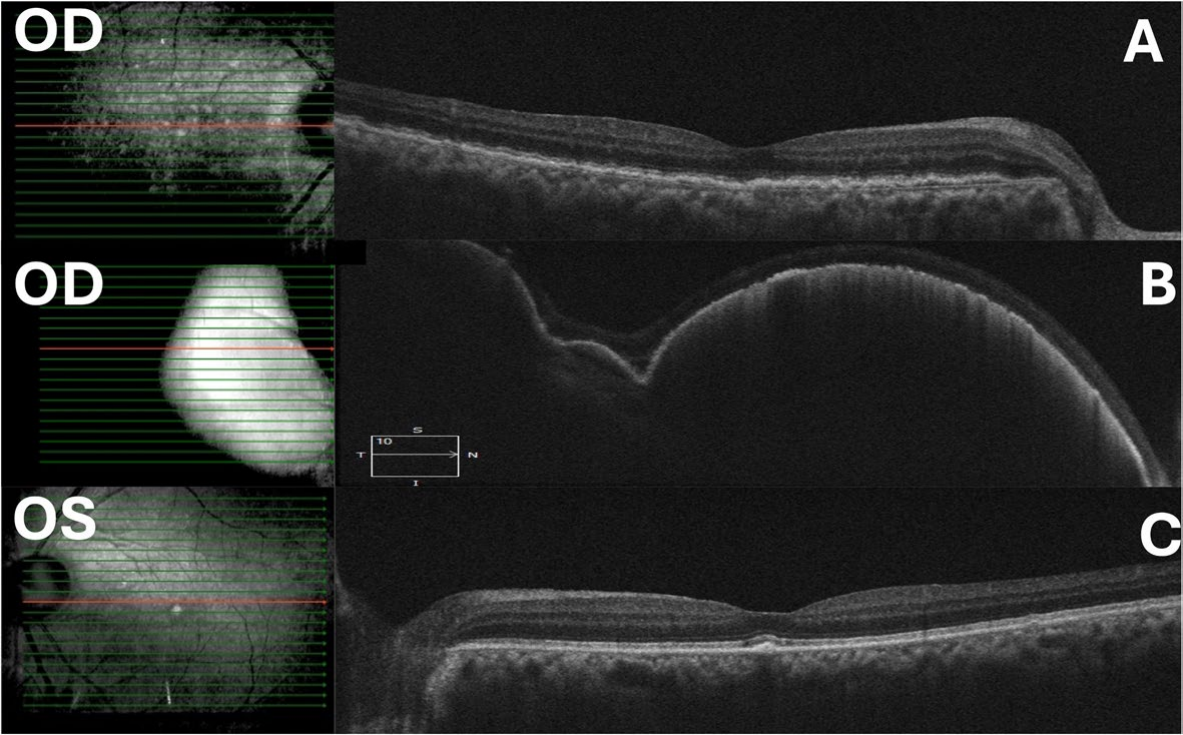
**A** SD-OCT of the RE through the posterior pole showed outer retinal and sub-RPE infiltrates. **B** Scan through the large temporal lesion showed homogenous, hyper-reflective deposits between the RPE and Bruch’s membrane. **C** SD-OCT of the LE showed few discrete RPE nodular infiltrates

Vitreous sample was sent for microbiological and cytological examination. Both were negative Post-operatively fundus examination of both the eyes showed clear media and cup disc ratio of 0.8. Right eye (RE) showed multiple foci of slightly elevated sub-retinal yellowish deposits with overlying retinal pigment epithelium (RPE) mottling with the largest lesion located temporal to the macula outside the inferior arcade (Fig. 1A). The left eye (LE) showed multiple dot like sub-retinal yellowish lesions with overlying pigment clumps in the mid periphery (Fig. 1B).Spectral domain optical coherence tomography (SD-OCT) scan in RE passing through the large temporal lesion showed homogenous, hyper-reflective deposit between the RPE and the bruch’s membrane (Fig. 2B) and the LE showed a few discrete RPE nodular infiltrates (Fig. 2C). This was highly suspicious of primary vitreo retinal lymphoma (PVRL).

**Fig. 3:**
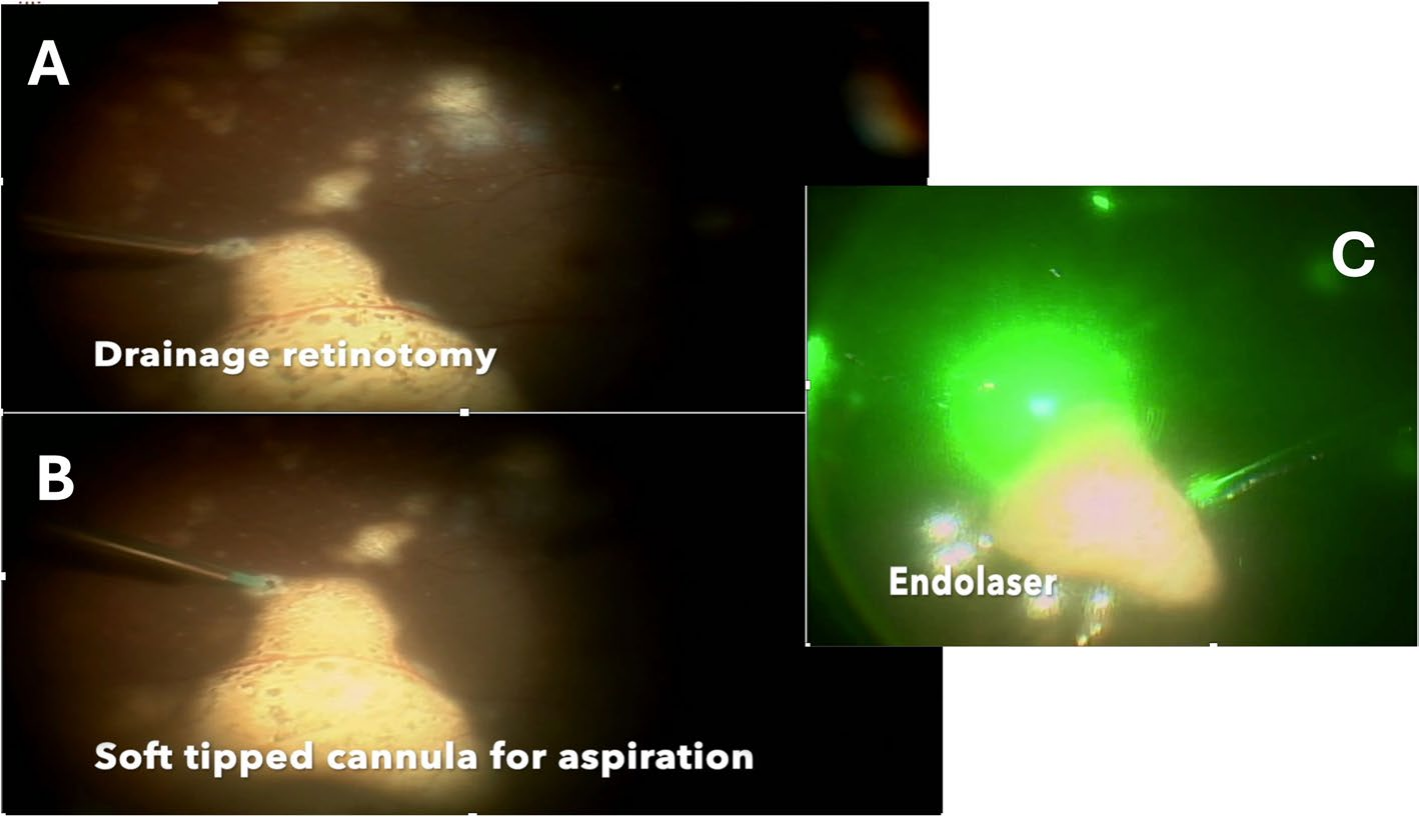
**A** Drainage retinotomy being created at the tip of the lesion in the RE. **B** Soft tipped cannula use to aspirate the material for examination. **C** Endolaser being applied to the retinotomy site

**Fig. 4:**
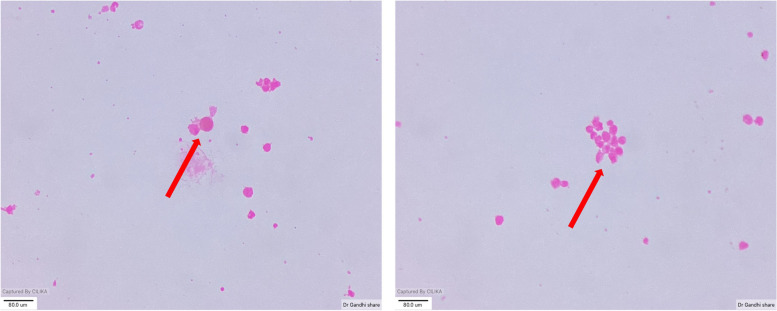
Cytology smear from the sample showed small and large lymphoid cells in a background of necrotic cells with some of the large cells showing prominent nucleoli

After an informed consent he underwent a sub-retinal biopsy in the RE using a 25 gauge pars plana vitrectomy system, a retinotomy was made using the diathermy at the superior edge of the large temporal lesion. The sub-retinal infiltrates were gently aspirated using a soft tipped backflush flute needle attached to a 2 ml syringe (Fig. 3B). This was followed by laser photocoagulation to the retinotomy site and 20% Sulfur hexafluoride(SF6) gas injection was used as a tamponade (Fig. 3). Cytological examination showed small and large lymphoid cells in a background of necrotic cells with some of the large cells showing prominent nucleoli (Fig. 4). A few macrophages and plasma cells were also noted. Microbiological examination showed no growth on staining and culture. Immunohistochemistry of the sample showed no IL-6 and IL-10 levels of 3.85. In view of the cytology report and a high IL-10/IL-6 ratio, a diagnosis of PVRL was made. Patient was sent for an opinion from a medical oncologist where he underwent fluorodeoxyglucose positron emission tomography (FDG PET) and cranial magnetic resonance imaging (MRI) scans. Both tests showed no evidence of systemic lymphoma.

**Fig. 5:**
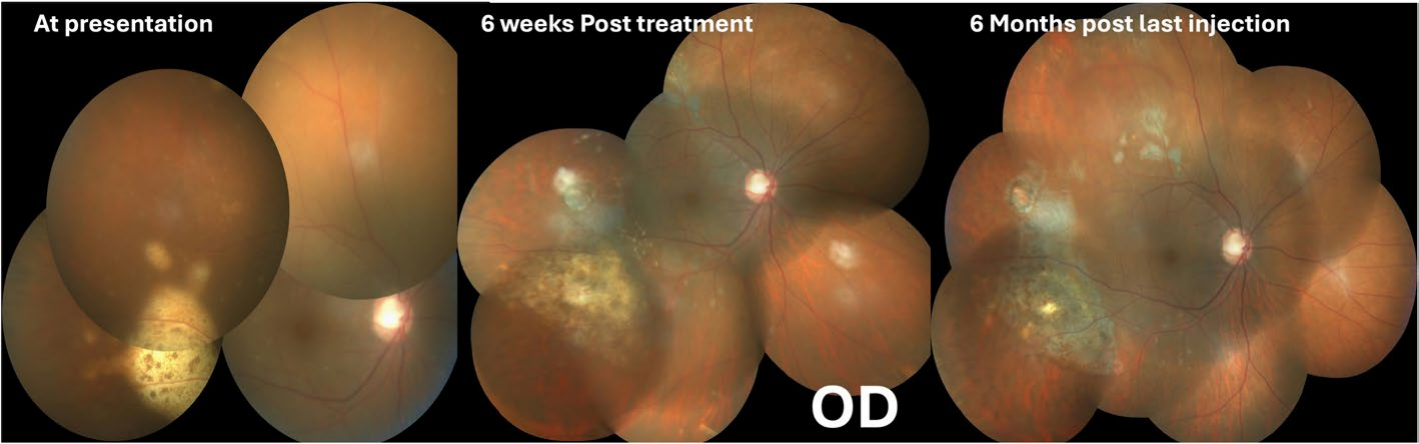
Timeline of events in the right eye. The lesion is healing at 6 weeks and appears scarred at 6 months post injection

Intravitreal Methotrexate (400μgms /0.1 ml)injections were started in both the eyes along with continuation of the anti-glaucoma medications twice a week. After receiving 7 and 6 injections in the RE and the LE respectively, the lesions were found to regress with pigmentation appearing over them. The height of the large temporal lesion in the right eye was noted to decrease on serial SD-OCT scans. (Fig.5) Due to the development of epithelial toxicity in the right eye and almost complete regression of lesions in both the eyes, a decision was made to switch both the eyes to weekly methotrexate injection regimen. Following this the RE received two and the LE received one weekly injection of methotrexate. Subsequently a monthly injection was given in both the eyes. At the 11th injection in RE and 8th injection in the LE, IL level analysis of aqueous humour was done. The IL-6 levels was 416.75 units and 388.34 units in the right eye and the left eye respectively and IL-10 was not detected.. Due to reversal of IL-10/IL-6 levels, he was continued on the monthly regimen for 3 more months.

**Fig. 6:**
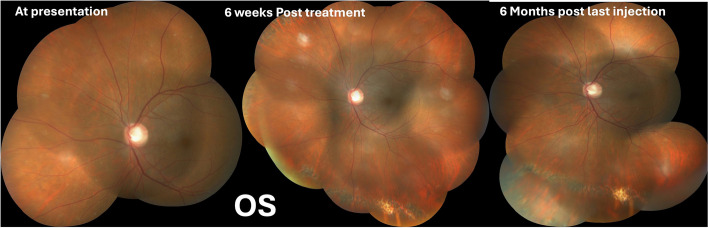
Timeline of events in the left eye. The lesion is healing at 6 weeks and appears scarred at 6 months post injection

Follow up visit at 8 months the RE had received 18 injections and the LE had received 15 injections. The BCVA was 6/6,N6 in both the eyes. IOP was well controlled on topical anti-glaucoma medications. Corneal epitheium and conjunctiva were within normal. Fundus examination showed regressed lesions in both the eyes with pigmentary alterations (Figs. 5 and 6). Subsequently the monthly injections of methotrexate were discontinued.

Patient was kept under a regular follow up and cranial MRI was repeated every quarter.

Last follow up at 18 months post presentation with 6/6,N6 vision and normal IOP in both the eyes. The most recent MRI, conducted 16 months after the initial presentation, was normal and showed no CNS involvement.

## Discussion

PVRL is a subset of the primary central nervous system lymphoma with ocular involvement [3]. It is characterized by monoclonal proliferation of lymphocytes. It is also known as primary vitreoretinal lymphoma (PVRL) or primary central nervous system lymphoma-ocular or ophthalmic variant (PCNSL-O) in literature [4, 5, 6, 7]. It is an extranodal non-Hodgkin, diffuse large B cell lymphoma. Very rarely, PIOLs may have T cell origin. It is a very rare ocular neoplasm with high morbidity and mortality. The disease is bilateral in 80% of the individuals and is asymmetric in presentation [7]. Bilateral vitreous haze with or without subretinal or sub RPE infiltrates with overlying RPE changes(leopard spot pigmentation) are common clinical features of PVRL [8].

PVRL a common mimicker of uveitis as it often presents with vitritis which tends to respond topical and oral corticosteroids. This causes an undue delay in the diagnosis and management of PVRL. As in our patient who had been diagnosed to have intermediate uveitis elsewhere and treated with long term corticosteroids with multiple recurrences [9]. This adds to secondary complications such as complicated cataract and secondary glaucoma.

Our patient underwent cataract surgery and IOL implantation in both the eyes at one week gap and was treated with topical anti-glaucoma medications. He had a past history of COVID-19 infection treated with high dose corticosteroids in the intensive care unit. Due to recent history of an intraocular surgery in both the eyes and a history of immunosuppression he was diagnosed to have endophthalmitis due to the presence of vitritis and subretinal yellowish exudates. Though our patient had no history of ocular pain or redness at the time of presentation which went against a diagnosis of endophthalmitis, but it was possibly due to the patient still being on topical steroids post cataract surgery. Clinically he was suspected to have fungal endophthalmitis and thus treated empirically with pars plana vitrectomy(PPV) and intravitreal antibiotics and antifungal. However the microbilogical and cytology report was negative as the patient was being treated with oral steroids for intermediate uveitis and we know that steroids have a lymphocytic effect that could be long lasting [5]. However the advantage of doing PPV was that the media had cleared thus making an OCT possible post operatively. SD-OCT passing through the yellowish sub retinal lesions showed characteristic OCT features of nodular mixed intraretinal, subretinal infiltration with large sub-RPE infiltrates strongly suggestive of PVRL. It was subsequently confirmed on sub retinal biopsy and cytological examination. IL-10 to IL-6 ratio also went in favour of PVRL. Although the reported sensitivity and specificity of this ratio in a large case series by Wang et al. [10] were 0.88 and 0.85 respectively. Therapy was initiated as per the Hadassah-Hebrew University protocol described by Frenkel et al. [11], but had to be modified due to the epithelial toxicity secondary to frequent methotrexate injections.

This case highlights the fact that when dealing with patients in older age group one should keep masquerades in mind. PVRL is an important differential in any middle aged or elderly patient presenting with bilateral recurrent vitritis. Our patient initially diagnosed as intermediate uveitis elsewhere was misdiagnosed as endophthalmitis due to the preceding history of intraocular surgery and immunosuppression with sub retinal yellowish deposits perceived as exudates on clinical examination. This case also highlights the importance of doing an early sub retinal biopsy after a false negative vitreous biopsy in patients treated with long term oral corticosteroids but having a high index of suspicion of PVRL on SD-OCT finding. This helps to confirm the diagnosis and treat the patient early causing remission of the neoplasm.

## Data Availability

No datasets were generated or analysed during the current study.

## Availability of data and materials

N/A.
